# Key Aspects of the Immunobiology of Haploidentical Hematopoietic Cell Transplantation

**DOI:** 10.3389/fimmu.2020.00191

**Published:** 2020-02-14

**Authors:** Susanne H. C. Baumeister, Benedetta Rambaldi, Roman M. Shapiro, Rizwan Romee

**Affiliations:** ^1^Division of Pediatric Hematology-Oncology, Boston Children's Hospital, Boston, MA, United States; ^2^Department of Pediatric Oncology, Dana-Farber Cancer Institute, Boston, MA, United States; ^3^Harvard Medical School, Boston, MA, United States; ^4^Division of Hematologic Malignancies, Dana-Farber Cancer Institute, Boston, MA, United States; ^5^Bone Marrow Transplant Unit, Clinical and Experimental Sciences Department, ASST Spedali Civili, University of Pavia, Brescia, Italy

**Keywords:** immunobiology, haploidentical, stem cell transplantation, NK-cells, graft-vs.-leukemia

## Abstract

Hematopoietic stem cell transplantation from a haploidentical donor is increasingly used and has become a standard donor option for patients lacking an appropriately matched sibling or unrelated donor. Historically, prohibitive immunological barriers resulting from the high degree of HLA-mismatch included graft-vs.-host disease (GVHD) and graft failure. These were overcome with increasingly sophisticated strategies to manipulate the sensitive balance between donor and recipient immune cells. Three different approaches are currently in clinical use: (a) *ex vivo* T-cell depletion resulting in grafts with defined immune cell content (b) extensive immunosuppression with a T-cell replete graft consisting of G-CSF primed bone marrow and PBSC (GIAC) (c) T-cell replete grafts with post-transplant cyclophosphamide (PTCy). Intriguing studies have recently elucidated the immunologic mechanisms by which PTCy prevents GVHD. Each approach uniquely affects post-transplant immune reconstitution which is critical for the control of post-transplant infections and relapse. NK-cells play a key role in haplo-HCT since they do not mediate GVHD but can successfully mediate a graft-vs.-leukemia effect. This effect is in part regulated by KIR receptors that inhibit NK cell cytotoxic function when binding to the appropriate HLA-class I ligands. In the context of an HLA-class I mismatch in haplo-HCT, lack of inhibition can contribute to NK-cell alloreactivity leading to enhanced anti-leukemic effect. Emerging work reveals immune evasion phenomena such as copy-neutral loss of heterozygosity of the incompatible HLA alleles as one of the major mechanisms of relapse. Relapse and infectious complications remain the leading causes impacting overall survival and are central to scientific advances seeking to improve haplo-HCT. Given that haploidentical donors can typically be readily approached to collect additional stem- or immune cells for the recipient, haplo-HCT represents a unique platform for cell- and immune-based therapies aimed at further reducing relapse and infections. The rapid advancements in our understanding of the immunobiology of haplo-HCT are therefore poised to lead to iterative innovations resulting in further improvement of outcomes with this compelling transplant modality.

## Introduction

Allogeneic hematopoietic cell transplantation (HCT) remains a curative approach for many patients with malignant and non-malignant hematologic indications (1). However, timely availability of a suitable HLA-matched sibling donor (MSD) or adequately HLA-matched unrelated donor (MUD) remains a significant challenge in providing access to HCT. The likelihood of finding an optimal donor varies significantly among racial and ethnic groups with the chances of finding an appropriate donor ranging from 75% for whites of European descent to 16% for blacks of South or Central American descent (2). Although most candidates for HCT will have a donor or cord blood unit considered suitable (HLA-matched or minimally mismatched), even single allele mismatches negatively impact patient outcomes after HCT (3). Additionally, proceeding with an unrelated donor is a time- and cost-consuming process that can result in delay or suboptimal timing of HCT.

**Figure 1:**
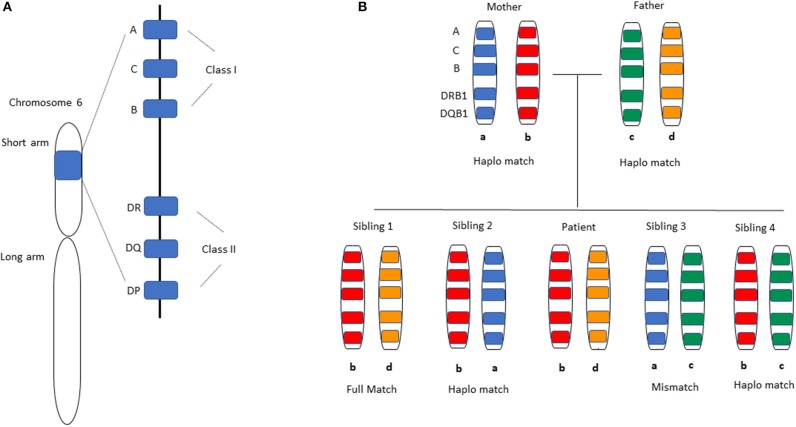
HLA-matching in Haplo-HCT. **(A)** Distribution of HLA alleles on chromosome 6. All HLA alleles exist on the short arm of the chromosome, specifically 6p21.3. The classical HLA classification system is used clinically for matching donors and recipients in the transplant setting. HLA class I alleles -A, -B, and -C are expressed on all nucleated cells and display antigen to CD8^+^ T-cells, while HLA Class II alleles -DR, -DQ, -DP are expressed on antigen-presenting cells and initiate a response by CD4^+^ T-cells. Not shown are the non-classical HLA Class I alleles -E, -F, -G, -H, -J that are also present on the same chromosome arm. **(B)** A representative inheritance pattern of HLA alleles is demonstrated. For a patient with HLA allele distribution b and d as shown in the middle, each sibling has a 25% chance of being a full match based on inheritance of the same maternal (b) and paternal (d) alleles as the patient. Each sibling has a 50% chance of being a haploidentical match by virtue of having inherited one identical allele (b) from the parents. The likelihood of having inherited neither of the parental alleles that were inherited by the patient is 25% (complete HLA-mismatch).

In contrast, haploidentical donors are available for >95% of patients in need of HCT (4). Biological children, parents, siblings, and frequently more distant family members who share one haplotype potentially qualify as donors (Figure 1). They can be readily identified and are typically available and motivated to donate bone marrow (BM) or peripheral blood stem cells (PBSC) to their family member in a timely fashion. This is particularly beneficial when unexpected events delay or expedite the need for HCT. Moreover, haploidentical donors can readily be tested in situations where there is concern for an underlying familiar predisposition syndrome and are typically available for a repeat stem cell collection, donor lymphocyte infusion or other cell therapeutic approaches which may be indicated if post-transplant complications such as graft failure, relapse, or infectious complications arise. Finally, if the selected family member had a poor stem cell mobilization for a PBSC graft or the optimal graft composition was not achieved then a different family member can be approached to serve as a haploidentical donor.

Historically, haploidentical HCT (haplo-HCT) was associated with high rates of graft vs. host disease (GVHD) and graft failure (5–7). With the introduction of efficient T-cell depletion (TCD) of the graft (8), haplo-HCT became feasible from a GVHD perspective. However, TCD led to an imbalance between host and donor T cells resulting in high rates of graft failure. This imbalance was overcome with the use of T-cell depleted “megadose” stem cell grafts (9, 10). Since then, nuanced *ex vivo* approaches to optimize the immunological composition of haploidentical grafts have been developed as outlined in this review.

A major milestone in promoting the wide-spread use and cost-efficient accessibility of haplo-HCT, including in resource-poor countries, was reached with the use of high-dose post-transplant cyclophosphamide (PTCy) to achieve *in vivo* attenuation of T cell alloreactivity (11). A different strategy using Granulocyte-colony stimulating factor (G-CSF) mobilized bone marrow grafts with extensive immunosuppression has been similarly feasible (12). In addition, a special emphasis is being placed on using natural killer (NK) cells to harness both innate and adaptive immunity in haplo-HCT. NK cells are uniquely regulated by activating and inhibitory receptors and can mediate a critical graft-vs.-leukemia (GVL) effect, also referred to as NK-cell alloreactivity, without mediating GVHD (13–15).

These approaches have contributed to a surge in the use of haplo-HCT in recent years (16). Furthermore, dramatic advances in the field of adoptive immune cell transfer have been applied to the haplo-HCT platform whereby donors could be readily approached for additional cell collections to enhance immunity against infections and relapse (17, 18). As haplo-HCT evolves to refine and establish its role in the field of transplantation, it is critical to examine the immunobiological properties unique to haplo-HCT and the effect of *ex vivo* or *in vivo* graft manipulation on the immunological content and trajectory of immune reconstitution.

## Challenges of the Hla-Barrier in Haplo-Hct

**Figure 2:**
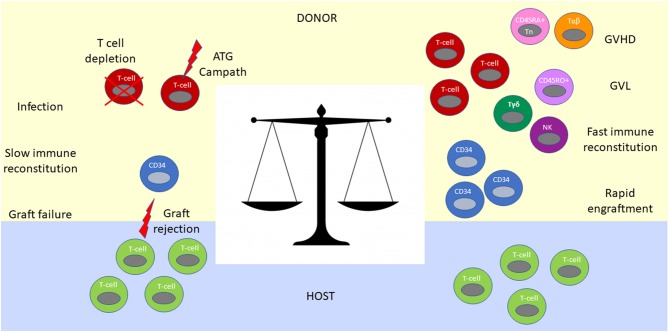
Immunological balance determines outcomes after haplo-HCT. The graft contains CD34^+^ and CD34^−^ hematopoietic cells. CD34^+^ progenitor and stem cells are required for engraftment and reconstitution of the bone marrow after transplantation into the host. T cells in the graft facilitate neutrophil engraftment, immune reconstitution, post-transplant infectious immunity and exert GVL effect **(Right)**. However, without an *ex vivo* (T cell depletion or CD34 positive selection) or *in vivo* (ATG or Campath) T cell depletion strategy, they mediate prohibitively severe GVHD **(Right)**. In contrast, extensive T cell depletion from the graft results in an immunologic imbalance between residual host and donor T cells favoring graft rejection **(Left)**. Extensive T cell depletion of the graft also results in slow immune constitution, infections and poor GVL control. To achieve an optimal immunologic balance, novel graft manipulation approaches selectively deplete T cells involved in GVHD (CD45RA^+^ T cell and αβ- T cell depletion strategies), while maintaining beneficial immune cells such as NK cells and γδT cells in the graft.

Early trials of T-cell-replete haplo-HCT were associated with poor outcomes due to a high incidence of GVHD and graft rejection, resulting in ~10% long-term survival (5–7, 19, 20). In the setting of grafting across a haploidentical HLA barrier, ~2% of donor T cells mediate alloreactive reactions resulting in GVHD while residual host T cells mount host-vs.-graft responses leading to graft rejection (21–23). The ability to overcome the problem of GVHD despite the large HLA-disparity in haplo-HCT was first demonstrated by Reisner and colleagues with the successful transplantation of children with severe combined immunodeficiency (SCID) using T-cell depleted haploidentical grafts which differed at three major HLA loci (8). However, when this approach was extended to other indications in which a patient's underlying immune system is generally functional, the minimal T-cell content in the graft resulted in unopposed host-vs.-graft rejections and a high rate of graft failure. The latter was mediated by recipient anti-donor T lymphocyte precursors that survived the conditioning regimen (22, 24, 25), as well as anti-donor HLA antibodies (26) (Figure 2).

A second breakthrough that paved the way toward the broad application of haplo-HCT was the use of “megadose” grafts, targeting the infusion of a stem cell product containing on the order of ≥10 × 10^6^/kg CD34^+^ hematopoietic stem cells while retaining the threshold dose of ≤4 × 10^4^/kg T cells established in the SCID patients (9, 10, 27, 28). The underlying immunologic effect of megadose grafting was attributed to tolerance induction of host anti-donor cytotoxic T cell precursors by donor CD34^+^ stem cells or by CD34^+^ derived regulatory immune cells endowed with a “veto”-effect in a TNFα mediated mechanism (29, 30). Intensified myeloablative conditioning (MAC) with 8 Gy total body irradiation (TBI), thiotepa, rabbit anti-thymocyte globulin (ATG) and fludarabine (replacing cyclophosphamide after 1995) (31) to eliminate host T cells, followed by G-CSF mobilized megadose T-cell depleted PBSC grafts (initially using soybean agglutination and erythrocyte resetting and later immunomagnetic selection of CD34^+^ HSCs) without any additional post-transplant immunosuppression was refined over the years (28, 32). This approach ultimately demonstrated primary engraftment in 95% of patients with acute leukemia (*n* = 104), with 6 of 7 patients who initially experienced graft failure engrafting successfully after second transplantation. Although acute and chronic GVHD were largely prevented, a significant non-relapse mortality (NRM) of 36.5% was observed largely owing to post-transplant infections (27 of the 38 patients died of infectious complications) and substantial relapse risk. The 2-year event-free survival (EFS) probability among patients receiving transplantation in any complete remission (CR) was 47%, while the EFS for those transplanted in relapse was 4% (27).

Despite the tremendous advances toward clinical feasibility of haplo-HCT, these early studies embodied the challenge of achieving a sensitive immunologic balance during transplantation across haploidentical HLA-barriers. This challenge is reflective of the need for extensive T-cell depletion and immunosuppression to control GVHD on the one hand, and facilitation of engraftment, immune reconstitution, protection from infections, and prevention of relapse on the other (Figure 2). This conundrum has fueled the iterative improvement of modulating immunity in the context of haplo-HCT as outlined below.

## Current HAPLO-HCT Platforms

### *In vivo* Haplo-HCT Strategies With Unmanipulated Stem Cell Grafts

#### Post-transplantation Cyclophosphamide (PTCy)

Post-transplantation high-dose cyclophosphamide (PTCy), when administered in a specific time-frame after graft infusion, efficiently attenuates alloreactive T cells from both donor and host and prevents GVHD and graft rejection. This immunological effect of PTCy was first observed in the 1960s in animal models of allogeneic skin grafts whereby cyclophosphamide administration within a window of up to 4 days after grafting delayed rejection (33). Subsequent preclinical studies defined the role of PTCy in the setting of allogeneic HCT and showed the benefits of its use with respect to engraftment and GVHD (34–36). Importantly the concurrent immunosuppression of T cells with cyclosporine or steroids interfered with PTCy-tolerogenic effects (37, 38), indicating that high proliferative rates are critical for the PTCy immunomodulatory mechanism (39).

Initial mechanistic studies based on murine skin allografting models attributed the PTCy-effect to the selective depletion of alloreactive T cells. Based on these hypotheses, the presumed depletion was dependent on the heightened cytotoxic sensitivity of newly primed and highly proliferative alloreactive T cells (particularly CD4^+^ T cells) at the peak of anti-host and anti-donor T cell expansion, aided by a favorable balance between effector T cells and regulator T cells (Tregs) as well as an additional intrathymic clonal deletion of alloreactive T cell precursors (40–44). Suppressive immune cells were only felt to have an adjunct role in maintaining tolerance (45, 46). However, recent work by Kanakry and colleagues formally tested the putative immunologic mechanisms (selective destruction of alloreactive T cells, intrathymic clonal deletion of alloreactive T cells and induction of suppressor T cells) in dedicated murine PTCy haplo-HCT models (47). These studies suggest that PTCy reduces CD4^+^ T cell proliferation but does not eliminate alloreactive T cells and instead functionally impairs the T-cell response to alloantigens and induces the rapid and preferential recovery and expansion of regulatory T cells (Treg). Treg resistance to PTCy is based on their differential expression of aldehyde dehydrogenase (ALDH) (48). Evidence for the importance of the role of Tregs after PTCy is exemplified by the development of severe and fatal GVHD in the context of Foxp3^+^ Treg depletion, as well as additional data showing that Tregs are required for PTCy-mediated protection against GVHD (49). Studies in thymectomized mice also suggested the dispensability of the thymus in this process (47). Advances in this active field of preclinical and clinical study are poised to further elucidate and facilitate adaption of the PTCy platform for different clinical scenarios. Increasing experience with this platform and the potential for PTCy-mediated bi-directional tolerance induction also lends itself to further exploration of this approach in the setting of combined solid organ and bone marrow transplantation (44).

**Figure 3:**
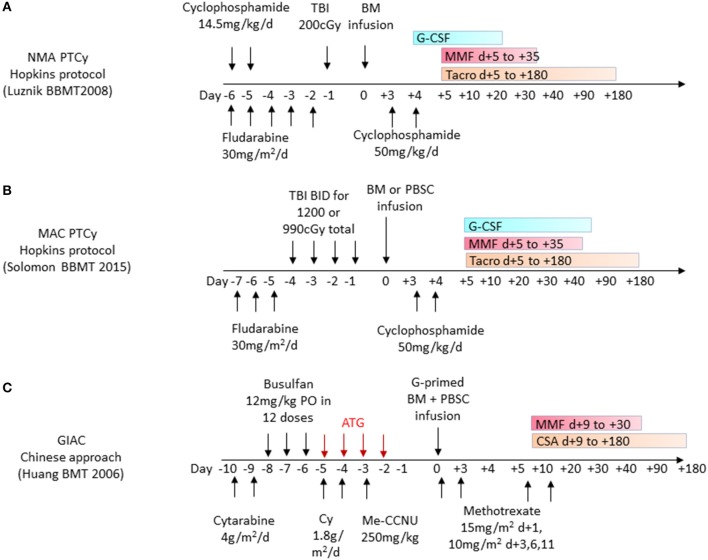
Frequently used haplo-HCT regimens. **(A)** Non-myeloablative (NMA) conditioning with administration of post-transplant cyclophosphamide (PTCy) as part of the Hopkins protocol for haplo-related donor HCT uses cyclophosphamide 50 mg/kg/day on days +3 and +4 and additional GVHD prophylaxis with oral MMF and tacrolimus (Tacro) starting on day +5. **(B)** Myeloablative conditioning (MAC) protocol with administration of post-transplant cyclophosphamide 50 mg/kg/day given on days +3 and +4 and additional GVHD prophylaxis with oral MMF and tacrolimus starting on day +5. **(C)** GIAC haplo-HCT protocol using a combination of G-CSF primed bone marrow (BM) and peripheral blood stem cells (PBSC) administered after a conditioning regimen including ATG on days −5 to −2. GVHD prophylaxis includes short-course Methotrexate in addition to MMF and cyclosporine (CSA).

**Figure 4:**
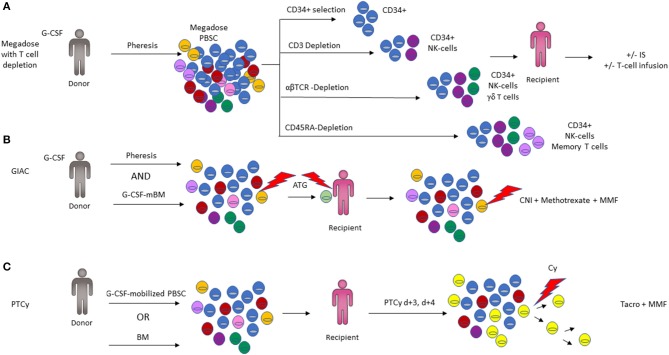
Comparison of the three major haplo-HCT platforms. **(A)** Four different *ex vivo* T-cell depletion protocols are shown, with the resulting cell composition in the graft. CD34-positive selection preferentially isolates the hematopoietic stem and progenitor fraction required for engraftment with minimal immune cell content (top panel). Depletion of CD3^+^ T-cells results in a graft composed predominantly of CD34^+^ and NK cells (2nd panel). Depletion of αβ-T cells depletes T cells involved in mediating GVHD but retains beneficial immune cells such as NK cells and γδ- T cells in the graft. CD45RA-Depletion removes naïve T cells including cells responsible for alloreactivity and GVHD, while retaining memory T cells including cells vital for immunity against infections (3rd panel). Additional immunosuppression (IS) and/or infusion of T-cell subsets may be employed post-transplant to optimize engraftment (3rd panel). **(B)** Representation of the GIAC protocol indicating a G-CSF primed bone marrow (BM) and peripheral blood stem cell (PBSC) graft, with ATG targeting T-cells derived from both the donor and recipient. GVHD prophylaxis with methotrexate, a calcineurin inhibitor (CNI), and MMF targets residual T cells (3rd panel). **(C)** Post-transplant cyclophosphamide (PTCy) functionally impairs actively proliferating recipient and graft derived T cells while favoring Treg recovery (color of cells corresponds to Figure 2; Tregs are depicted in bright yellow).

The first clinical study of unmanipulated haplo-HCT with PTCy was conducted in the setting of non-myeloablative (NMA) conditioning with administration of PTCy at 50 mg/kg on day +3 and an added immunosuppressive regimen of mycophenolate mofetil (MMF) and tacrolimus starting on day +4 in 13 patients (50) (Figures 3A, 4C). Subsequent prospective clinical trials, administering PTCy either on day +3 or on days +3 and +4, demonstrated rates of graft failure and GVHD comparable to those reported with reduced intensity conditioning (RIC) HLA-matched sibling and MUD HCTs with a trend toward a lower risk of extensive chronic GVHD among recipients of two doses of PTCy (50). These studies paved the way for the increased investigation and clinical use of haplo-HCT with PTCy (Figure 3B).

#### GIAC Approach (G-CSF-Mobilization, Intensified Post-transplant Immunosuppression, ATG and Combination of PBSC and BM Allografts)

The GIAC approach using T-cell replete haploidentical grafts was pioneered at Peking University (12, 51). This approach uses ATG as part of the conditioning regimen, which affects recipient T cells and facilitates engraftment. Owing to its long half-life, it also exerts effects on donor T cells and therefore impacts GVHD and post-transplant immunity. The graft consists of a combination of G-CSF-primed bone marrow and PBSC, thereby combining the advantages of both elements. PBSC grafts contain 2–3-fold higher CD34^+^ cells and a log-fold higher T cell dose than are typically contained in a steady-state bone marrow graft (52), and this has been shown to accelerate engraftment and decrease the relapse rate (Figures 3C, 4B).

The higher T cell dose in PBSC grafts adversely affects chronic GVHD but not acute GVHD rates in unrelated donor HCT (53). Multiple mechanisms may contribute to why acute GVHD rates are not drastically higher despite the high T cell dose. These include preferential dendritic cell mobilization and T cell polarization (54, 55), attenuating effects on costimulatory molecules such as CD86 on APCs and CD28 on CD4^+^ T cells (56, 57), as well as IL-10 mediated T-cell suppression by monocytes (58). Several studies underscored the benefit of utilizing G-CSF mobilized bone marrow, leading to less acute and chronic GVHD while maintaining engraftment rates comparable to PBSC (59) and have attributed these effects to differences in cytokine milieu, T-cell polarization and T-cell hypo-responsiveness (60–62).

In the initial study of 171 patients using GIAC, most of whom had ALL, AML, or CML, all patients engrafted with sustained full donor chimerism. The rates of leukemia-free survival and incidences of grade II-IV acute GVHD and extensive chronic GVHD were comparable to MUD HCT (12, 53). A prospective multicenter study of AML patients has demonstrated that transplant outcomes with the GIAC strategy have also been comparable to MSD HCT (63). Although a modified approach using G-CSF primed haploidentical bone marrow and extensive GVHD prophylaxis has also been applied in Europe (64), the GIAC strategy has been used most extensively in China and therefore patients transplanted with this strategy represent a large cohort of haploidentical transplants HCT treated to date (65).

### Haploidentical Hct With *ex vivo* T Cell Depletion or Anergy Induction Strategies

#### CD34^+^ Cell Selection

The establishment of procedures for the *ex vivo* removal of T cells from the graft in the late 1970s by Reisner, O'Reilly and colleagues, represented a tremendous breakthrough toward the feasibility of utilizing haploidentical donors. In the initial approach, T cells were eliminated from the bone marrow by first rosetting with sheep red blood cells followed by differential soybean agglutination of residual T lymphocytes in the non-rosetting population. This yielded an un-agglutinated fraction containing a high proportion of colony-forming cells without any detectable T cell alloreactivity, and abrogated lethal GVHD in murine models (66, 67). This strategy was applied in the first clinically successful haploidentical HCT of an infant with AML, leading to sustained hematopoietic engraftment without GVHD until relapse occurred 11 weeks after HCT (68). Three infants with SCID were also treated with this approach of whom 2 had sustained engraftment and none developed GVHD (8).

CD34^+^ selection, now in wide-spread use in TCD transplants, was first introduced in the 1990s. This process utilizes a CD34^+^ directed antibody coupled to immunomagnetic beads to positively select CD34^+^ cells and isolate them over a magnetic column. This effectively eliminates all other immune cells, including T-, B-, NK-cells, dendritic cells and monocytes from the graft (69, 70). This process was further refined with the use of micromagnetic beads, which had the advantages of high purity selection via attachment to single cells and safe infusion into patients (71). Aversa and colleagues of the Perugia group pioneered a novel haploidentical HCT platform incorporating an intensified conditioning regimen to eliminate host T cells and administering megadose T cell depleted grafts without additional post-grafting immunosuppression (28, 72). Handgretinger et al. tested this approach with G-CSF mobilized megadose PBSC grafts in 39 children lacking suitable donors and observed low rates of GVHD, but significant relapse and treatment-related mortality (TRM) (73). Investigators from Perugia further evaluated this system in adults with high-risk leukemia using megadose haplo-HCT, demonstrating 91% primary engraftment and low rates of GVHD without post-transplant GVHD prophylaxis (27) (Figure 4A, top panel).

#### CD3^+^ Cell Depletion

To improve post-transplant immune reconstitution, control of infections and prevention of relapse, further iterations of immunomagnetic graft engineering were developed (74). This included the elimination of CD3^+^ T cells and CD19^+^ B cells using a negative immunomagnetic selection method to deplete these subsets from the graft. Stem cells, NK cells, myeloid precursors, monocytes, and other progenitor cells important for engraftment are preserved (75). This strategy maintains innate immunity in the graft while removing CD3^+^ T cells capable of inducing GVHD. Depletion of CD19^+^ B cells was introduced to reduce the risk of post-transplant lymphoproliferative disease (PTLD) (73) and GVHD (76). While the depletion of donor B-cells reduces the risk of PTLD, it does not address PTLD arising from residual host B cells. Instead, this can be addressed with the inclusion of rituximab or Campath (but not the T cell directed agents ATG or OKT3) into the conditioning regimen (77, 78). Several centers established CD3^+^/CD19^+^ depletion as a feasible approach for patients lacking a suitable donor, with excellent primary engraftment and reduced rates of GVHD correlating with the remaining CD3^+^ cell/kg content of the graft. However, the low OS rate of 31% remains primarily attributable to infections and relapse, suggesting that further improvement of TCD haplo-HCT is needed (79, 80) (Figure 4A, second panel).

#### αβT-Cell/B-Cell Depletion

With emerging recognition of γδT cells (81), a yet more sophisticated approach was developed for GVHD prevention. In contrast to αβ-T-cell receptor (TCR) expressing T cells, γδ-TCR expressing T-cells are not implicated in mediating GVHD (82) but do exhibit important functions characteristic of innate immune recognition and anti-tumor effects (83, 84). These cells represent 1–20% of all CD3^+^ circulating T lymphocytes in human peripheral blood and the majority of resident T cells in skin and mucosa. Their TCR heterodimer consists of a γ and δ chain encoded by a limited repertoire of V, D, and J gene segments. The two major Vδ1 and Vδ2 subsets are distinguished based on their TCRδ composition. Whereas, Vδ1^+^ cells are typically associated with a Vγ1/2/3/5/8 chain, the majority of Vδ2^+^ T cells express an invariant TCR harboring Vγ9. The Vγ9δ2 TCR is expressed by the majority of peripheral γδ T cells, whereas γδ T cells including other Vδ elements are predominantly enriched at epithelial surfaces and the skin (81, 84). Analogous to NK cell biology, γδT cells are fine-tuned by activating and inhibitory receptors and recognize conserved non-peptide antigens that signal potential danger or cellular stress. The activating receptor NKG2D is broadly expressed in γδT cells and functions synergistically with the γδ-TCR as a costimulatory receptor (85, 86).

γδT cells have heterogenous functions, ranging from protection against intra- and extracellular pathogens or malignant cells to modulation of the immune response and tissue homeostasis. They contribute to pathogen clearance through the production of granulysin, defensins, and cytotoxic effector molecules such as perforin and granzymes (84). γδT cells secrete proinflammatory cytokines involved in protective immunity against viruses, intracellular pathogens (TNF-α and IFN-γ), extracellular bacteria, fungi (IL-17), and extracellular parasites (IL4, IL5, IL13), and have been shown to exhibit lytic activities against leukemia, lymphoma and carcinoma cells (87–89). Indeed, increased γδT-cell numbers after allogeneic HCT were associated with a lower incidence of infections and improved disease-free survival (DFS) in several studies (90–92).

In a pediatric trial using αβ-T cell/B-cell depleted haplo-HCT, γδ-T cells were the predominant T-cell population in the initial weeks after transplantation, specifically expanded in response to CMV reactivation, and displayed cytotoxicity and degranulation when challenged with primary leukemia blasts *in vitro* (93). These effects were increased after exposure to zoledronic acid, suggesting that the anti-leukemic capacity of γδ-T cells could further be enhanced (94). Outcomes with the αβ-T cell/B-cell depleted haplo-HCT approach in which no additional GVHD prophylaxis was employed appear promising both in children with malignant (95) and non-malignant conditions (96), and when compared with MUD and MMUD HCTs in a retrospective analysis of children transplanted for acute leukemias (97). However, the high incidence of viral infections reported by some groups highlights the potential to further improve *ex vivo* T-cell depletion strategies (98) (Figure 4A, third panel).

#### CD45RA-Depletion

As our understanding of T cell differentiation status and phenotype has become increasingly sophisticated, so have approaches to tailor graft composition further (99, 100). αβ-T cells exist as distinct subsets that can be differentiated by cell surface phenotype: naïve (T_N_), stem cell memory (T_SCM_), effector (T_E_), effector memory (T_EM_), and central memory (T_CM_). The CD45RA^+^CD62L^+^ T_N_ subset is antigen inexperienced, has a more diverse TCR repertoire than memory T cells and clonally expands following T cell priming to execute short-lived effector functions. They ultimately differentiate into memory subsets, which is associated with downregulation of CD45RA and upregulation of CD45RO. Studies in mouse models demonstrated that T_N_ mediated severe GVHD, whereas T_CM_ induced milder GVHD and T_EM_ were devoid of GVH activity (101–105). Importantly memory T cells transferred infectious immunity and GVL activity in these models (106).

Based on the premise that elimination of T_N_ from the graft could significantly reduce GVHD while maintaining pathogen- and tumor-specific immunity, Bleakley and colleagues developed a novel graft-engineering strategy using immunomagnetic beads coupled to a monoclonal Ab targeting CD45RA. The latter antigen is expressed on all T_N_, but absent on Treg, T_CM_ and most T_M_ (107). This strategy was initially studied in patients with high risk hematologic malignancies undergoing MSD HCT, utilizing a 2-step selection procedure with a CD34^+^ selection of stem cells (a minor subset of which expresses CD45RA) followed by depletion of CD45RA^+^ cells from the CD34^−^ fraction. This study demonstrated engraftment in all patients (*n* = 35), prompt immune recovery without excessive rates of infection or relapse and low chronic GVHD, but interestingly no reduction in acute GVHD although the latter was readily steroid-responsive (108).

Clinical results with CD45RA-depletion in the context of haplo-HCT are so far limited. A study of 17 pediatric patients with high risk hematologic malignancies using a RIC conditioning with total lymphoid irradiation (TLI) but without TBI or serotherapy, administered a CD34^+^ selected PBSC product on day 0, followed by a CD45RA-depleted PBSC product which had been collected the following day, and ultimately a donor NK cell product administered on day +6 with the use of Sirolimus or MMF post-transplant. Rapid neutrophil engraftment and memory T-cell reconstitution was observed, without any infectious deaths and with 76.5% of patients alive at a median of 225 days after HCT. Grade III-IV acute GVHD and chronic GVHD were seen in 3 and 6 of 17 patients, respectively (109). In a second small study, 5 children with combined immunodeficiency and chronic viral infections received a combination of a CD34^+^ selected product and the CD45RA-depleted fraction of the CD34-negative product with post-HCT prophylaxis consisting of Cyclosporine and MMF. One patient died with graft failure. In the 4 engrafted patients, viral infections cleared within 2 months after HCT and an early T cell response against viral pathogens was documented in 2 patients (110). Further studies will be needed to further define the role of this approach in haplo-HCT (Figure 4A, bottom panel).

#### *Ex vivo* Induction of T Cell Anergy With CTLA-4Ig

An early strategy to minimize T-cell alloreactivity by interfering with the priming of alloreactive T cells in haplo-HCT was explored in a pediatric trial. This involved collection of patients' peripheral blood mononuclear cells (PBMC) prior to the start of myeloablation and a 36-h *in vitro* incubation of the recipient cells with non-mobilized donor bone marrow in a mixed lymphocyte reaction (MLR) setting in the presence of CTLA-4Ig, a fusion protein which inhibits priming of alloreactive T cells by inhibiting costimulatory signaling between the B7 protein family (CD80/CD86) on APCs and CD28 on T cells (111). This reduced the frequency of T cells recognizing alloantigens of the recipient while preserving responsiveness to alloantigens of other persons. In this trial of 11 evaluable patients most of which had persistent disease at the time of HCT, 5 were alive and in CR at 4.5–29 months after transplant with 3 patients developing steroid-responsive acute GVHD of the gut only. There were no deaths attributable to GVHD (112). However, this approach has not been explored further.

#### Photodynamic Purging of Adoptive T Cell Therapy Following TCD Haplo-Hct

A different approach to augment the TCD graft with an adoptive T cell therapy product devoid of alloreactive T cells is a process termed photoallodepletion. Prior to G-CSF mobilization of the PBSC graft, donors undergo non-mobilized leukapheresis to obtain T cells. Donor T cells are then incubated with recipient PBMC in an MLR in the presence of TH9402, a photosensitizer similar to rhodamine. T cell activation in the MLR, which occurs selectively in the alloreactive T cells but spares Tregs and pathogen-specific T cells, is associated with P-glycoprotein pump inhibition leading to mitochondrial accumulation of TH9402 in alloreactive T cells (113, 114). Subsequent activation of TH9402 with visible light leads is then selectively toxic to and eliminates alloreactive T cells via an oxidative damage mechanism (115). Early results from a clinical trial in which patients received the photodynamically allodepleted T-cell product subsequent to a CD34^+^ selected graft appear promising (116).

## Role of Innate Immunity in HAPLO-Hct

NK-cells are an important component of the innate immune system providing protection against infectious pathogens and cancer. Recent studies have elucidated that human NK cell diversity is much broader than the traditional distinction via CD56^bright^ and CD56^dim^ subsets reflective of differentiation stage and cytotoxic potential. The ability of NK cells to differentiate into long-lived cells with memory capacity (117) and the discovery of non-NK innate lymphoid cells has highlighted the complexity and potential roles of innate immune cells after HCT (118, 119). NK cells have potent anti-leukemia effector capacity, respond to viral infections via release of toxic granules, and facilitate engraftment without mediating GVHD. This is particularly important in the setting of heavily T cell-depleted grafts or T-cell directed post-transplant immunosuppression and has inspired a rich field of investigation to augment NK cell immunity in the context of HCT to develop leukemia-directed NK-cell based cellular therapies.

**Figure 5:**
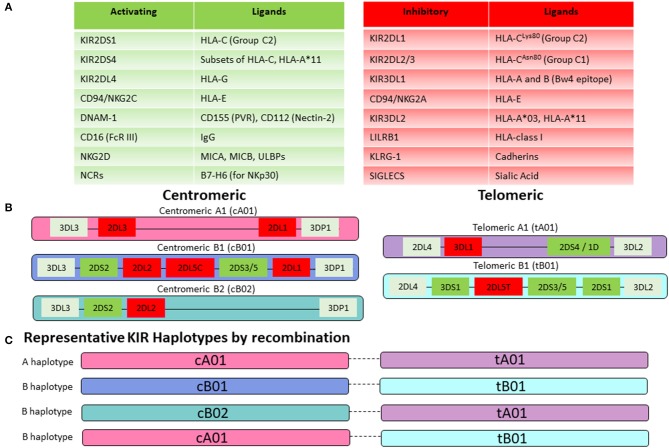
NK-cell receptor repertoire. **(A)** NK-cell activity is mediated by a balance of activating and inhibitory signaling. Key activating receptors and their corresponding ligands are listed in the green table, while inhibitory receptors are displayed in the red table. **(B)** KIR genes are highly polymorphic and organized in centromeric and telomeric motifs with structural variation that creates multiple gene content haplotypes. Group A haplotype motifs are characterized by fewer genes and predominantly those encoding for inhibitory KIRs. In contrast, Group B haplotype motifs are enriched for activating KIRs. **(C)** Centromeric and telomeric motifs are paired together to generate either a KIR A haplotype (composed of centromeric and telomeric A motifs) or a KIR B haplotype (containing at least one centromeric or telomeric B motif). Representative KIR haplotypes by recombination are shown here. Prominent linkage disequilibrium has been noted within the centromeric and telomeric motifs but not between them, suggesting that pairing occurs by recombination between the centromeric and telomeric regions.

NK-cell activity is governed by the balance of a system of activating and inhibitory NK cell receptors (120). Activating signals are provided by receptors such as NKG2D, CD94/NKG2C and Natural Cytotoxicity Receptor (NCRs) including NKp30, 44, and 46 and by activating killer-cell Ig-like receptors (KIR). NKG2D recognizes MHC-class I related stress-ligands that can be upregulated by tissues in response to infection, inflammation, DNA-damage, and malignant transformation (121), while CD94/NKG2C binds to the non-classical HLA-E molecules and senses overall HLA-Class I expression on cells (Figure 5A). NK cells utilize a unique process to balance tolerance to self under steady state conditions with the ability to mediate an immune response to pathogens or malignant cells. This is referred to as NK-cell education or licensing (122), is in large part regulated by inhibitory KIR receptors and impacts NK-cell alloreactivity in the setting of haplo-HCT and allogeneic NK-cell therapies (123).

KIRs are either activating or inhibitory based on their structure. The KIR nomenclature incorporates the number of extracellular Ig-like domains (two in KIR2D vs. three in KIR3D) and whether the KIR contains a long or short tail (KIR2DL vs. KIR2DS). KIRs are further numbered in order of their discovery within their structural group (KIR2DL1 vs. KIR2DL2). KIRs with long tails are generally inhibitory (with exception of KIR2DL4) and KIRs with short tails function as activating receptors according to presence or absence of immunoreceptor tyrosine-based inhibitory motifs (ITIMs) (124). There is tremendous variability within the KIR repertoire owing to a high degree of polymorphism among individual KIR genes as well as their organization and recombination within haplotypes (Figures 5B,C) (125). An individual's genetic KIR repertoire is determined by the inherited composition of centromeric and telomeric A and B haplotypes (Figure 5B). Group A haplotypes contain fewer genes and predominantly those encoding for inhibitory KIRs. Additionally, the activating KIR2DS4 gene exist as an inactive deletion variant, termed KIR1D in the majority of Caucasians, leaving the framework gene KIR2DL4 as the sole receptor on this haplotype with any activating function (126, 127). In contrast, Group B haplotypes are enriched for activating KIRs. Two groups of KIR haplotype can be assigned based on the combination of the centromeric and telomeric motifs. Presence of a centromeric or telomeric B-haplotype constitutes a KIR B haplotype whereas the combination of a centromeric and a telomeric A-haplotype results in a KIR A haplotype (Figure 5C). Although more than 50 different haplotypes have been described, there are 11 common haplotypes derived by reciprocal recombination, which collectively account for 94% of Caucasian haplotypes examined by Jiang et al. (128). Distribution of a KIR gene in the centromeric or telomeric region of chromosome 19q13.4 is further thought to impact KIR-mediated regulation of NK-cell activity (129). Additionally, KIR-cell surface expression at the protein level may vary substantially from the inherited KIR gene profile. This is attributable to the fact that KIRs are stochastically expressed on NK cells and each NK cell may therefore display a different cell-surface profile of inhibitory or activating KIRs (130). For the most accurate prediction of NK-cell alloreactivity between haploidentical donor and recipient, KIR-genotyping alone is insufficient and determination of the KIR phenotype (by flow cytometry) should also be pursued.

**Figure 6:**
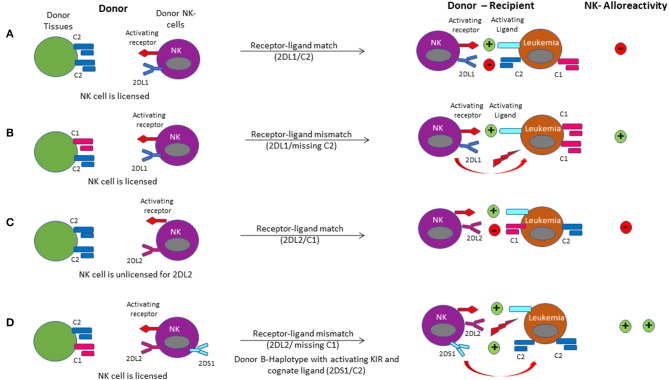
NK cell alloreactivity in haplo-HCT is demonstrated via the different models of receptor-ligand mismatch. **(A)** The donor-derived NK cell is licensed when its KIR2DL1 receptor had been engaged by expression of its cognate C2 ligand in the donor environment. Upon infusion of the licensed NK cell into the recipient, a leukemia cell expressing the C2 ligand will not activate the NK cell due to a receptor-ligand match. **(B)** A receptor-ligand mismatch occurs when the donor-derived NK cell is licensed, but the recipient does not express the C2 ligand (missing ligand). Provided that it is further driven by stimulation through activating receptors, this results in activation of the licensed donor NK cell upon infusion into the recipient, leading to a graft-vs.-leukemia effect. **(C)** If the donor does not express the appropriate class I ligand for its KIR receptor (HLA and KIR segregate independently), the donor NK cell is unlicensed. In this case, donor NK cells are accustomed to a missing ligand. They may be activated when encountering strong activating signals (like activating cytokines) or be further inhibited when encountering the inhibitory ligand in the recipient. **(D)** Licensing of the NK cell for the C1 ligand occurs in the donor. Upon transplant into the host, the missing C1 ligand coupled with binding of the activating ligand with the activating receptor on the NK cell results in alloreactivity. Binding of the activating KIR receptor KIR2DS1 to the C2 ligand on the target leukemia cell enhances NK cell alloreactivity.

The majority of inhibitory KIRs recognize classical (HLA- A, B, and C) or non-classical HLA-class I molecules (HLA-G) as their cognate ligands (Figure 5A) (131). KIR genes are located on chromosome 19 whereas HLA-genes are located on chromosome 6. KIR and HLA genes therefore segregate independently, and an individual may or may not express the cognate HLA-ligand for any given KIR. This forms the basis for the concept of “education” or “licensing” of NK-cells, which allows NK-cells to maintain self-tolerance under physiologic conditions, while retaining the ability to mount an immune response (132). When NK cells encounter the matching HLA-class I ligand for their inhibitory KIR (based on the requisite germline inheritance of the appropriate HLA and KIR genes and their expression patterns on individual NK cells), they are considered “educated” or “licensed” and refrain from an attack on healthy tissues under steady state. However, when NK cells are accustomed to this inhibitory signal and subsequently encounter a cell that does not express the appropriate KIR-ligand (“missing ligand”), this situation renders them functional to mount an effector response, if the target also expresses stress-ligands that trigger activating NK-cell receptors (133). A missing ligand may be encountered on malignant cells due to HLA-class I downregulation, or HLA-mismatched allogeneic transplantation such as haplo-HCT, when the recipient does not express the corresponding HLA-ligand (Figure 6). NK-cells are considered “unlicensed” when they do not encounter the matching HLA-class I ligand for their given inhibitory KIR. Due to the lack of exposure to their corresponding ligand, unlicensed NK-cells are “un-educated” and hyporesponsive at steady state rather than being triggered by self-tissues lacking the ligand (134). Unlicensed NK cells require a higher threshold for activation. However, in the absence of KIR inhibition, they can mediate higher levels of effector function when they receive strong stimulatory signals under inflammatory conditions (such as CMV infection or in the posttransplant setting) or when triggered for antibody-dependent cellular cytotoxicity (ADCC) (135, 136). Given that NK cells may surface-express variable combinations and densities of inhibitory KIRs, NK-cell education occurs on a continuum along which individual NK cells display graded levels of responsiveness based on their KIR profile and engagement of cognate HLA-class I ligands (122, 137, 138).

Since the model of NK-cell alloreactivity in the context of mismatched HCT was first proposed, a number of studies have evaluated its clinical impact (139). For the interpretation of HCT studies evaluating the role of NK-cell alloreactivity it is critical to consider the definition of the KIR-mismatch model employed in each study (131, 140) (Figure 6). The “KIR ligand-ligand mismatch model” is based on the hypothesis that the presence of the corresponding HLA-ligand prevents NK-cell alloreactivity, whereas a missing ligand in the HCT recipient triggers NK cell alloreactivity. However, while this model accounts for HLA-class I mismatches, it does not consider KIR-genotype or phenotype. In contrast, the “KIR receptor-ligand mismatch model” accounts for the fact that a missing ligand is irrelevant if NK cells do not express the corresponding KIR for a mismatched HLA-class I ligand. Therefore, this model incorporates the HLA-ligand repertoire in the recipient as well as the donor KIR genotype and ideally phenotype. Other groups have employed the “KIR-haplotype model” which takes into consideration the presence or absence of a B-KIR haplotype in the donor, as a measure of enrichment for activating vs. inhibitory KIRs. Use of this model demonstrated a reduced risk of leukemia relapse when patients were transplanted from donors with centromeric B-haplotypes (141–143). Similarly, more recent approaches have focused on the predicted overall degree of inhibitory and activating KIR-KIR ligand interactions between the recipient and potential donors with a highly variable KIR repertoire. This allows for selection of an optimal donor, even when the transplant recipient's HLA-class I repertoire is such that all KIR ligands are expressed and a missing-ligand scenario is unachievable.

Ruggeri et al. first established that a NK-cell alloreactivity of the donor toward recipient (based on KIR receptor-ligand mismatch in the GVL direction and presence of alloreactive clones against recipient targets) lowered the AML relapse risk in the context of *ex vivo* depleted haplo-HCT (72). These results were subsequently consolidated in a larger cohort of 112 AML patients, where transplantation from a NK-cell alloreactive donor was associated with a significantly lower relapse rate (3% compared to 47%) when transplanted in complete remission and better EFS when transplanted in relapse (34% compared to 6%) or CR (67% compared to 18%) (144). Subsequent studies of sibling donor, unrelated donor (URD), and umbilical cord blood (UCB) donor sources have yielded variable results (14). Some studies showed no benefit or even inferior survival resulting from a mismatch in the KIR/KIR-ligand system. This may be partly related to the variable definition of KIR-mismatch models and transplant regimens used. In contrast, a large analysis in AML patients undergoing 9/10 or 10/10 URD employed an algorithm to predict the strength of inhibition between the ubiquitous KIR3DL1 and its ligand HLA-B and found that combinations with absent of weak inhibition were associated with significantly lower rates of relapse and overall mortality (145). The extent of T-cell depletion may also play an important role, since the presence of T cells in the graft affects NK cell reconstitution leading to lower KIR-receptor expression (146). Lastly, given that a KIR ligand-ligand mismatch implies an absence of a KIR ligand in the host that is present in the donor, it equates with the presence of a major HLA-class I mismatch. It is therefore not unexpected that such mismatch leads to significant T-cell alloreactivity and poor survival unless T-cell reactivity is minimized with methods such as TCD.

A retrospective analysis of 161 patients receiving TCD haploidentical allografts confirmed a beneficial role of NK cell alloreactivity. In the presence of KIR-receptor-ligand mismatches in the GVL direction, expression of activating KIR2DS1 or KIR3DS1 was associated with a significant reduction in NRM, largely owing to 50% reduction in infection rates (147). While much of the benefits of NK cell alloreactivity are reported for myeloid indications, a pediatric study of 85 patients undergoing TCD haplo-HCT showed that patients transplanted for ALL from a KIR B-haplotype donor had a significantly better EFS than those with KIR haplotype A donors. Additionally, a higher KIR B-content score (based on the number of centromeric and telomeric KIR B motifs) was associated with a significant reduction in relapse risk (148). Although limited by use of a KIR ligand-ligand model, a study of haplo-HCT with PTCy for various hematologic malignancies found that KIR-ligand mismatch was associated with a lower incidence of relapse and better PFS for patients transplanted in relapse but had no significant impact on those transplanted in CR (149). A growing ability to navigate the complexities of the KIR-system, such as recognition of varied strengths of inhibition among subtypes of inhibitory KIRs and its ligands resulting in discrete hierarchies of anti-leukemic cytotoxicity will aid in further revealing how donor selection based on KIR-compatibility may improve outcomes (145). While the beneficial effects of NK-cell alloreactivity are mostly documented in the context of *ex vivo* T cell-depleted haplo-HCT, the growing adaptation of T-cell replete haplo-HCT affords the opportunity to carefully study the role of NK-cell alloreactivity in these platforms.

## Immune Reconstitution After HAPLO-Hct

Transplant outcomes are directly related to the achievement of an acceptable restoration of the immune system. Several cell subsets play a key role in the protection toward infections and disease recurrence. In general, innate immunity recovers early after transplant and represents the first line of defense against pathogens. Specifically, monocytes followed by neutrophils and NK cells arise in the first month after transplant. Adaptive immunity mediated by T and B cell lymphocytes recovers later and is crucial for both immune tolerance maintenance and long-term protection against infections and disease relapse. T cell reconstitution can occur through two different mechanisms: thymus-independent T cell peripheral expansion of infused donor memory T cells and thymus-dependent *de novo* generation of donor T cells from donor hematopoietic progenitors (150).

**Figure 7:**
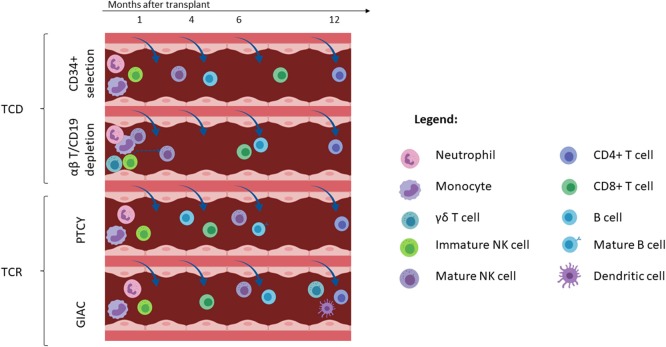
Immune reconstitution with different haploidentical transplant platforms. Only subsets which have been characterized by published primary data for each platform are included in this figure (e.g., data on dendritic cell reconstitution has only been described for the GIAC approach). Cells are depicted at the approximate time-point of reaching the lower range of normal. T-cell depleted (TCD) haploidentical transplant is associated with early recovery of neutrophils, monocytes, immature NK cells and rapid NK cell maturation which takes 6–8 weeks (top panel). Additionally, αβ-T cell/CD19 depletion is associated with early detection of γδT cells and mature NK cells that are infused with the graft infusion (2nd panel from the top). B cell recovery is delayed with CD19 depletion relative to CD34^+^ selection. T-cell replete (TCR) haploidentical transplant performed with post-transplant cyclophosphamide (PTCy) and GIAC protocols is associated with early reconstitution of immature NK cells (3rd panel from the top). It is also associated with earlier reconstitution of CD8^+^ T-cells than TCD protocols. The GIAC protocol is associated with delayed dendritic cell recovery (bottom panel). This figure was created using BioRender.com.

While the kinetics of immune reconstitution and its correlation with HCT outcomes are well-established in the setting of matched donor transplant, more studies are needed in the setting of haplo-HCT. Different donor sources do not represent the only cause of possible differences in immune reconstitution kinetics. Specific haplo-HCT platforms and GVHD prophylaxis approaches are also crucial factors to consider (151). As detailed above, two major haplo-platforms are currently used: T- cell replete haplo-HCT that use an *in vivo* T-cell depletion with ATG or PTCY, and TCD haplo-HCT in which the graft is *ex vivo* manipulated with a CD34-positive selection or a T-cell negative selection. Here, we review the immune reconstitution of different blood cell subsets after different types of haplo-HCT (Figure 7).

### Monocytes

Monocytes are the first immune subset to recover after HCT. Rapid and robust monocyte CD14^+^ cell reconstitution has been correlated with the improvement of transplant outcomes in the setting of MSD (152) and UCB-HCT (153). Recently, a study by Turcotte and colleagues showed that higher absolute monocyte count (AMC) and higher classic monocyte subsets (CD14^bright^ CD16^−^) at day +28 were associated with a reduced risk of relapse and TRM, better 2-yr OS, and improved 2-yr PFS in a cohort of patients transplanted for different hematological malignancies using both RIC or MAC regimens and different stem cell sources (154). AMC was influenced by the graft origin, with a higher AMC found in UCB but no differences between BM and PBSC. However, no haplo-HCTs were included in this study. In a separate cohort of 144 patients treated with MAC conditioning for hematological malignancies, receiving a T-cell replete graft consisting of G-CSF-mobilized BM and PBSC from HLA-haploidentical or MSDs, the monocytes recovered rapidly, and the AMC was above the normal range starting from the first month to the first year after transplant. Both patient groups received GVHD prophylaxis with Methotrexate, Tacrolimus, MMF, and Cyclosporine with the addition of ATG in the haploidentical group (GIAC protocol). Monocyte reconstitution was comparable between recipients after HLA-matched and haplo-HCT on days +30, 90, and 180 after transplantation. None of the patient transplant characteristics impacted monocyte recovery in the multivariable analysis (155). Finally, in a pediatric cohort of 40 patients receiving TCD haplo-HCT using CD34 positive selection or CD3/CD19 cell depletion, monocyte expansion was rapid, reaching normal values for age within 30 days of transplant. Moreover, no differences in monocyte recovery were seen between different graft purification and conditioning intensity regimens (156).

### Neutrophils

Depending on the study, neutrophil engraftment is defined by the presence of more than 500 or 1,000 neutrophils/μL of blood and represents a crucial step in the early phase after transplant. Prolonged neutropenia is associated with severe infection and increased TRM (157). In the setting of a T-cell replete transplant, neutrophil recovery occurs quickly. With GIAC protocols, the median neutrophil engraftment was achieved at 14 days (range 9–25) (158, 159), whereas with the RIC PTCY platform using BM grafts and Tacrolimus and MMF GVHD-based prophylaxis, the median time to neutrophil recovery was 15 days (range 11–42) (11). For both protocols, patients received recombinant human granulocyte colony-stimulating factor (rhG-CSF) from day +6 or +4 to engraftment, respectively.

In the context of TCD HCT using the Perugia protocol with CD34^+^ selected megadose grafts, the median time to neutrophil recovery was 11 days (range 9–30) without G-CSF support (27). Studies using CD3/CD19 cell depletion in adult patients also showed rapid neutrophil recovery, with a median time of 12 days (range 9–50) without the addition of G-CSF (79, 80). Similar results were seen in a cohort of pediatric patients with acute leukemia undergoing MAC transplant. Specifically, patients in the αβ-T cell-depleted haplo-HCT had a faster neutrophil recovery compared to MUD, mismatch unrelated donors (mMUD), and those treated with Methotrexate and Calcineurin-inhibitors, with median time to neutrophil engraftment of 13 (range 6–23), 19 (range 9–46), and 20 days (range 10–120), respectively (97). All three groups received ATG during the conditioning for prevention of graft failure and GVHD, and none of the patients received G-CSF to accelerate neutrophil recovery. Taken together, these data show that haplo-HCT provides a comparable or even expedited neutrophil recovery compared to standard matched donor-HCT.

### Dendritic Cells

Dendritic cells (DCs) represent a rare population in the peripheral blood, accounting for 0.15–0.7% of mononuclear cells (160). In the context of T-cell replete haplo-HCT using the GIAC protocol, Wang and colleagues measured the frequencies of DCs and their subsets among white blood cells (WBCs) after haplo-HCT, including CD123^+^ plasmacytoid DCs (pDCs) and CD11c^+^ myeloid DCs (mDCs). Recipients had strikingly decreased proportions of DCs (0.49% vs. 0.27%, *P* = 0.025), mDCs (0.27% vs. 0.14%, *P* < 0.001), and pDCs (0.04% vs. 0.02%, *P* = 0.008) in the WBC compartment at ~180 days post-haplo-HCT compared to healthy subjects. Since, it was reported that primary human DCs were the most potent expander of the γδ T cell subset Vδ2^+^ (161), the authors also investigated whether the recovery levels of Vδ2^+^ T cells were associated with the DC content following transplantation. Bivariate correlation analysis showed that the proportion of mDCs, but not DCs and pDCs, in WBCs was significantly correlated with the recovery of Vδ2^+^ T cells after haplo-HCT. Specifically, slow recovery of mDCs was associated with a slow recovery of Vδ2^+^ T cells in this haplo-HCT setting (162).

Chang and colleagues also described a slower DCs recovery at +15 and 30 days after HCT compared to those in the HLA-matched recipients in another study (158). In their protocol, ATG was administrated only in the haplo-group. Indeed, it was described that ATG not only induced a tolerogenic phenotype in human DCs (163), but was also able to mediate a complement-mediated lysis of DCs (164). In summary, these findings may explain the delay in DC recovery in the setting of the haplo-HCT using the GIAC protocol. The kinetics of DC reconstitution in other haplo-HCT settings, remain to be fully characterized.

### Natural Killer (NK) Cells

Due to the need to perform an extensive T cell depletion in haplo-HCT, anti-tumor efficacy is largely dependent on the graft-vs.-leukemia effect exerted by NK cells that eradicate residual leukemic blasts surviving the preparative regimen (72, 165–167). In the haplo-HCT setting performed through the infusion of positively selected CD34^+^ cells, the first emergence of fully functioning, KIR alloreactive NK cells from hematopoietic progenitors may require at least 6–8 weeks, and therefore the benefit offered by their anti-leukemia effect is delayed (168–171). In the setting of αβ-T-cell/CD19 depletion, generation of NK cells from donor HSC takes ~8 weeks but circulating NK cells can be detected earlier after transplant due to infusion with the graft (172). Moreover, CMV reactivation in this setting was associated with an expansion of memory-like NK cells (NKG2C^+^, CD57^+^, KIR^+^) as early as 3 months after HCT (173). Surprisingly, in a pediatric comparison between TCD haplo-HCT performed with CD34 positive selection or CD3/19 negative selection, NK-cell recovery was faster in patients receiving PBSC from CD34^+^ positive selection in the first 4 months after transplant (156).

In the T-cell replete haplo-HCT setting using PTCY, Russo and colleagues described that donor alloreactive NK cells infused with the graft were killed by cyclophosphamide (174). This translated into a delay of NK recovery and maturation resulting from a profound reduction after cyclophosphamide administration following a robust proliferation of donor-NK cells in the early phase after graft infusion. The absence of aldehyde dehydrogenase (ALDH)-positive NK cells suggested that they were susceptible to cyclophosphamide cytotoxicity, and this was then confirmed using an *in vitro* assay of mafosfamide-induced cell death (174). On the other hand, Russo et al. reported an IL-15 peak in patient sera at day +15 after transplant that was associated with a progressive increase of NK cells expressing an immature phenotype (CD62L^+^, NKG2A^+^, KIR^−^) between day +15 and day +30 (174). The normal distribution of NK phenotypes was achieved only between 9 and 12 months after transplant, with a decrease of CD56^bright^, NKG2A, and CD62L expression and an increase of maturation markers (CD16, CD57, and KIR). KIR expression returned to normal levels around day +60, but NKG2A expression decreased only after 6 months. Interestingly, in this cohort of patients, there was no difference in PFS between patients with or without a predicted KIR alloreactivity, suggesting that the protective anti-tumor activity of NK cells is dampened after T-cell replete haplo-HCT using the PTCY platform (174).

Another group described the transient and predominant expansion of an unconventional subset of NK cells characterized by a specific phenotype: NKp46^neg/low^, CD56^dim^, CD16^neg^, CD94/NKG2A^high^ starting from the second week after transplant and maintained until the 7th week (175). This unconventional population retained its proliferative capacity and the ability to differentiate into the CD56^bright^ subsets (NKp46^+^, CD56^bright^, CD16^−^ cells) in response to IL-15 and IL-18. Despite the unconventional NK cells expressing a high level of activating receptors (NKG2D and NKp30), Granzyme-B and Perforin, they displayed a defective *in vitro* cytotoxicity highlighting again the need to improve NK reconstitution after PTCy haplo-HCT (175). Similar results were reported in the GIAC protocol in which early and higher expression of CD94/NKG2A was inversely correlated with KIR expression, and was associated with worse survival (176). The same group showed that NK cells from patients who developed GVHD had a lower expression of NKG2A, lower proliferative capacity and an increased rate of apoptosis, but retained their cytotoxicity after *in vitro* co-culture with the K562 cell line (177).

Finally, in contrast to TCD haplo-HCT, KIR-mismatch analysis between donor-recipient pairs when using only HLA and KIR genotyping without consideration of the KIR phenotype, was unable to predict post-transplantation outcomes in multivariate analyses in the setting of haplo-HCT using the GIAC protocol (178). However, it has been reported that KIR-driven NK cell alloreactivity is better predicted if donor KIR genotype is considered in conjunction with KIR cell surface expression (130). Moreover, in haplo-HCT using the GIAC protocol, the higher number of T-cells infused in the graft contributed to the high incidence of acute GVHD (178). This resulted in a need for increased immune suppression, thereby affecting NK alloreactivity.

### T Cells

Achievement of an acceptable T cell reconstitution after HCT represents a crucial goal and correlates with better transplant outcomes. Impairment of T cell reconstitution is more pronounced after T cell depletion (152). In the context of T-cell replete haplo-HCT using the GIAC protocol, CD3^+^ T cell counts were 125, 883, 1,163, and 1,308 cells/μL at 30, 90, 180, and 360 days after HCT, respectively (158). A lower median CD3^+^ T cell count was reported after NMA haplo-HCT using a BM graft with PTCy, Tacrolimus and MMF based GVHD prophylaxis, with 206 cells/μL at day 40 and 219 cells/μL at day 100 (179). On the other hand, CD3^+^ T-cell recovery was more rapid with 338 cells/uL at day +30 after MAC haplo-HCT using PBSC grafts with PTCy, MMF, and sirolimus GVHD-based prophylaxis (180).

In the setting of T-cell replete haplo-HCT with both GIAC and PTCy-based protocols, CD8^+^ T cells recovered earlier than CD4^+^ T cells (158, 181–183). Faster CD8^+^ T cell recovery at day +90 correlated with higher CD3^+^ cells in the graft but was not associated with a higher incidence of GVHD (184). The same studies highlighted that the recovery of CD4^+^ T cells was impaired for the whole first year after transplant, but failed to demonstrate a correlation between delay in CD4^+^ T cell reconstitution and NRM as was shown in the HLA-matched donor setting (185). Notably, in the GIAC experience the delay of CD4^+^ T-cell reconstitution was compensated by the proportional increase of the CD8^+^ T cell- and monocyte fractions, and the NRM was relatively low (19.5% in the haplo group vs. 17.4% for the matched-sibling donor cohort). This was likely due to patient care improvements, especially the management of CMV reactivation (158).

A retrospective EBMT registry study including both adult and pediatric patients undergoing haplo-HCT found an association between higher CD3^+^, CD4^+^, and CD8^+^ T-cell counts and better OS with less NRM (186). However, in the multivariable analysis only higher CD3^+^ and CD8^+^ T-cell counts correlated with lower NRM. No association was found between any of the T-cell, B-cell, or NK-cell subset counts with relapse-related mortality. In this study, the majority of patients were treated with TCD haplo-HCT using both CD34^+^ selection and CD3/19 depletion (186). In the context of αβ T-cell depleted haplo-HCT, CD3^+^, and CD3^+^/CD8^+^ T-cell recovery was slower compared to MUD or MMUD-HCT until 6 months after transplant (97). Recovery of CD4^+^ T cells was delayed only in the first 3 months and became even better at 1 year after haplo-HCT compared to MUD and MMUD. In this pediatric experience, haplo-HCT patients did not receive any additional pharmacological GVHD prophylaxis, whereas MUD and MUD HCT were performed using standard calcineurin-based GVHD prophylaxis and short-term methotrexate (97).

T memory stem cells (T_SCM_) represent a subset of early-differentiated human memory T cells with stem cell-like properties. T_SCM_ and naïve T cells (T_N_) both express naïve markers such as CD45RA, CCR7, and CD62L, but in distinction to T_N_ and similar to other memory subsets, T_SCM_ are characterized by CD95 expression. In the context of haplo-HCT using PTCy, two different groups elegantly showed that donor-derived T_SCM_ reconstitute early after transplant, representing the majority of both CD4 and CD8 T cells at day +8. At the polyclonal, antigen-specific, and clonal level, T_SCM_ lymphocytes were preferentially derived from differentiation of T_N_ infused within the graft, whereas most memory infused lymphocytes are purged by PTCy (182, 187).

### Regulatory T (Treg) Cells

Treg cells play a key role in the modulation of immune tolerance after HCT. Higher Treg content in the graft has been associated with better OS and lower aGVHD (188), whereas a reduced frequency of Tregs contributed to cGVHD incidence after matched-donor transplant (189). In the matched donor setting, Kanakry and colleagues showed that Treg, especially memory CD45RA-Treg, were preserved and recovered rapidly while conventional T (Tcon) naïve cells were reduced when PTCy was used as the sole method of GVHD prophylaxis (48). This was ascribed to the high levels of aldehyde dehydrogenase (ALDH), as the major *in vivo* mechanism of Cyclophosphamide resistance in the Treg population. In addition, murine studies demonstrated the importance of Tregs for GVHD reduction in the context of the PTCy-based GVHD prophylaxis (49).

In the T-cell replete haplo-HCT setting using PTCy, naïve Tregs increased after cyclophosphamide administration. This was attributed to the lower Ki67 levels compared to the memory subsets at day +3. In addition, Tregs exhibited a lower proliferation profile compared to Tcons, suggesting a lower susceptibility to PTCy in the haploidentical setting (182). This effect seems to be enhanced when PTCy is combined with sirolimus instead of a calcineurin inhibitor (180). Cieri et al. showed an expansion of CD25^+^CD127^−^FoxP3^+^ Tregs early after transplant, relative to the donor leukapheresis content and to the quantity in healthy subjects. Interestingly, patients who did not experience acute GVHD had a higher percentage of circulating Tregs at day +15 compared to patients who developed acute GVHD (180). Notably, the ability of Sirolimus to boost Treg reconstitution has also been reported outside of the PTCy platform. Indeed, Peccatori and colleagues reported an expansion of Treg after haplo-HCT using a combination of ATG, sirolimus and MMF as GVHD prophylaxis (190). Moreover, in the Baltimore experience with a cohort of patients undergoing MAC haplo-HCT using PTCy, MMF, and tacrolimus-based GVHD prophylaxis, Tregs achieved normal donor levels at all time-points examined (day +30, +90, +180, and +365) (181). Finally, in haplo-HCT using the GIAC protocol, patients with a higher day +30 percentage of naive Treg, defined as CD4^+^CD25^+^CD45RA^+^, had a significantly lower incidence of grades II–IV acute GVHD (191). This highlights the importance of reaching a satisfactory Treg reconstitution for the achievement of immune tolerance after haplo-HCT.

### γδ T Cells

γδ T cells combine conventional adaptive immunity features with innate-like MHC-independent tumor recognition (192). In healthy donors the majority of circulating γδ T cells expresses the Vδ2 chain, whereas the minority expresses the Vδ1 chain. The former subgroup is able to recognize non-peptide phosphoantigens and to perform direct killing of tumor cells (193). The Vδ1 γδ T-cell subgroup on the other hand is associated with control of CMV infection and also retains antitumor activity (194). Both subgroups play a key role in the setting of haplo-HCT because they do not induce GVHD but can exert immunological surveillance. In patients undergoing αβ-TCD haplo-HCT, γδ-T cells were the predominant T-cell subset for the first 2–3 weeks after transplant (91.5% of CD3^+^ lymphocytes), while αβ T cells became the most prevalent population at 1 month (93). Moreover, patients had a higher proportion of γδ-T cells, especially the Vδ2^+^ subset for the first 3 months. However, CMV reactivation (but not infection with other viruses) was associated with an expansion of Vδ1 γδ-T cells (93). Interestingly, the authors showed that zolendronic acid was able to potentiate Vδ2^+^ killing against leukemia blasts after *in vitro* culture, indicating that the cytotoxicity was dependent on phosphoantigen recognition and providing a rationale for the development of future clinical trials to boost the γδ T anti-tumor effect (93). The same group tested the *in vivo* ability of zolendronic acid (ZOL) to enhance γδ T-cell recovery and function, by administrating the drug to pediatric patients undergoing αβ-TCR/CD19 depleted haplo-HCT. An induction of Vδ2-cell differentiation paralleled by increased cytotoxicity of both Vδ1 and Vδ2 cells against primary leukemia blasts was associated with ZOL treatment. Patients given three or more ZOL infusions had a better probability of survival in comparison to those given one or two treatments (86% vs. 54%, respectively, *p* = 0.008), suggesting that ZOL infusion promotes γδ T-cell differentiation and cytotoxicity and may influence the outcome of patients in this transplant setting (94).

### B Cells

B cell recovery occurs late after HCT. B cells are almost undetectable during the first and second months and normal values are only reached around 12 months after transplant (195). In the setting of NMA haplo-BMT using PTCy, MMF and Tacrolimus as GVHD prophylaxis, B cells were undetectable until day +28. Recovery of B cells started from week 5 with an immature CD38^bright^ CD10^+^ Ki-67 negative phenotype, suggesting that the increase in B-cell number was not due to the homeostatic proliferation of transferred B cells but to *de novo* generation (196). Maturation of B cells was characterized by different expression of both transitional (T) markers CD5 and CD21: T0 (CD5^−^CD21^−^), T1 (CD5^+^CD21^−^), T2 (CD5^+^CD21^+^), and the CD5^−^CD21^+^ subset. Starting at week 9, mature B cells (CD38^dim^ CD10^−^) began to increase with a naïve phenotype (IgD^+^, IgM^+^). Overall, B cell maturation took 6 months to complete in the setting of a T-cell replete PTCy-based haplo-HCT (196).

With haplo-HCT using the GIAC protocol, median B cell counts did not differ from HLA-matched HCT at any of the time points examined (158). In an analysis comparing CD34 positive selection and CD3/CD19 cell depletion, B cells reconstituted more rapidly in the former group (156). Furthermore, recovery of B cells after αβ T cell-depleted haplo-HCT was delayed for the first 6 months compared to a cohort of patients transplanted with a MUD or MMUD using standard calcineurin-based GVHD prophylaxis. However, this is at least in part attributable to the fact that in the αβ T-cell depletion setting, patients received one dose of Rituximab as part of the conditioning regimen in order to prevent post-transplant lymphoproliferative disorders (97).

## Relapse and Immune Evasion Mechanisms After HAPLO-Hct

Recent data has highlighted the critical role of the immune system in the control of myeloid leukemia after HCT and elucidated our understanding regarding the immunologic mechanisms underlying relapse after haplo-HCT. Work by Vago and colleagues revealed that a substantial proportion of AML and MDS relapses after haplo-HCT are attributable to acquired uniparental disomy of chromosome 6p (copy-neutral loss of heterozygosity eliminating the incompatible HLA alleles without decreasing the overall level of expression of HLA class I molecules). This was shown to result in loss of the mismatched HLA molecules on leukemia cells and immune escape from leukemia control exerted by haploidentical donor T cells via the major histocompatibility mismatch (197). The maintained overall expression of class I molecules in this study also evaded activation of NK-cell mediated anti-leukemic responses which could potentially be based on a newly missing ligand to an inhibitory KIR receptor (197). Clinical suspicion for an immune evasion phenomenon was first raised when patients relapsing after haplo-HCT had discrepant findings in host chimerism monitoring between short-tandem-repeat amplification but not HLA typing (198). Recognition of this leukemia escape mechanism has therapeutic importance for patients who are candidates for subsequent haplo-HCT in whom a different donor is available who is mismatched for the HLA haplotype retained in the relapsed leukemic cells and/or is predicted to mediate NK-cell alloreactivity based on the newly missing KIR-ligand. The development of routine diagnostic methods is expected to facilitate this (198). Importantly, ~30% of relapses after haplo-HCT are attributable to this mechanism of the elimination of the incompatible HLA alleles irrespective of the GVHD prophylaxis or platform used to control T-cell alloreactivity (190, 199, 200).

**Figure 8:**
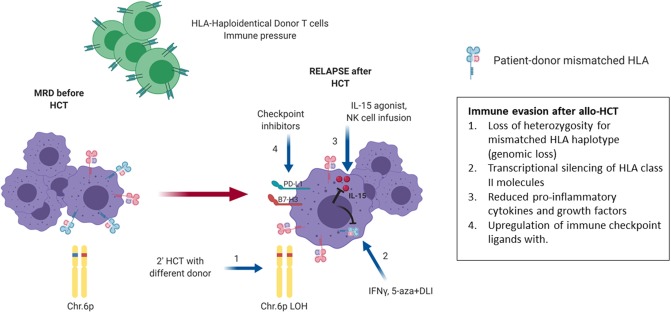
Mechanisms of relapse post haploidentical HCT. Late relapse after haploidentical allogeneic transplantation can be driven by a number of immunologic mechanisms as shown. Under the immune pressure of graft-vs.-leukemia (GVL) via HLA mismatch in a haploidentical environment, loss of heterozygosity for the mismatched HLA allele is a mechanism of escape from immune surveillance and relapse (1). Another mechanism involves transcriptional silencing of HLA class II molecule, thereby reducing T-cell mediated GVL. This effect can be partially reversed in the presence of immunomodulatory molecules such as IFN-y or the epigenetic regulator 5-azacitidine (5-aza) (2). Modification of the tumor microenvironment via suppression of release of mediators that promote GVL is another mechanism used by relapsing leukemia cells, which may be partially reversed via administration of IL-15 agonists and NK cell infusions that promote the secretion of proinflammatory cytokines (3). An additional common mechanism of relapse involves the emergence of T-cell exhaustion with associated upregulation of PD-L1 and other inhibitory receptors. The latter may be reversed through administration of checkpoint inhibitors (4). Blue arrows indicate possible therapeutic strategies to overcome the different mechanisms of immune evasion. MRD, minimal residual disease; LOH, loss of heterozygosity; Chr, chromosome; DLI, donor lymphocyte infusion. This Figure was created using BioRender.com.

To identify other drivers of post-HCT relapse Toffalori et al. analyzed transcriptional signatures specific for post-transplant AML relapses (201). This study demonstrated deregulation of the costimulatory interface between donor T cells and host leukemia cells, with loss of costimulatory interactions and enforcement of inhibitory ones (PD-1/PDL-1) as evidenced by both changes in leukemic cells and donor T cells (Figure 8). Additionally, the study documented downregulation of surface expression of HLA class II molecules on leukemia cells due to the downregulation of the HLA class II regulator CIITA (201). Patients with AML relapse after HCT were found to have a higher proportion of BM—infiltrating T cells expressing inhibitory receptors (IR) compared to patients remaining in CR. The exhausted BM-T cell phenotype was associated with a restricted TCR repertoire, impaired effector functions and leukemia-reactive specificities. Furthermore, early detection of severely exhausted BM-memory stem T cells predicted relapse (202). Interestingly, IR-positive T cells infiltrating the BM of AML patients at relapse displayed a greater ability to recognize matched leukemic blasts after *in vitro* expansion compared with their IR-negative counterparts. This suggest that IR expression marks lymphocytes enriched for tumor specificity whose activity could be unleashed with therapeutic check-point blockade, although innovative targeted strategies will be required to avoid exacerbation of GVHD in the HCT context (202).

## HAPLO-Hct as a Platform for Post-Transplant Immune Therapies

**Figure 9:**
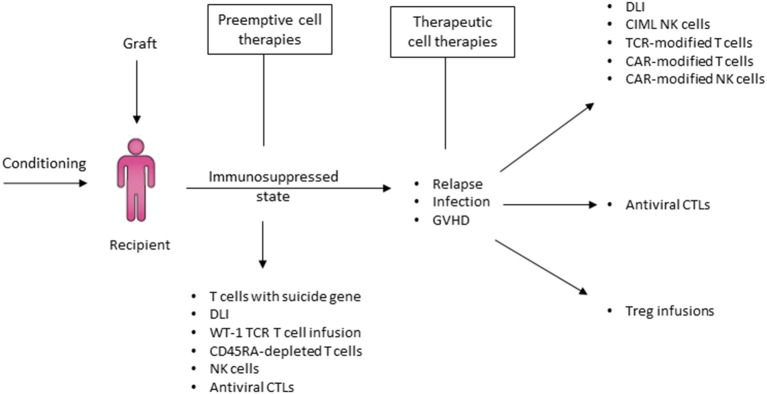
Haplo-HCT offers a platform for post-transplant immune therapies to prevent and treat relapse. In the context of a haploidentical transplant, there are several options to administer cellular therapies in order to address relapse, infection and GVHD either pre-emptively or therapeutically. In the event of a relapse, enhancing GVL effect using cellular therapy that either relies on the haploidentical mismatch between donor and recipient or gene-modified donor immune effector cells T cells are potential options. Donors haploidentical to the recipient may also readily serve as a source of cells for the production of CAR-T or CAR-NK. In the event of significant viral infection post relapse, administration of antiviral cytotoxic T-cells may promote viral clearance without increasing the risk of GVHD. Finally, Treg infusions may be utilized to treat GVHD. DLI, Donor lymphocyte infusion; CIML NK cells, Cytokine-induced memory-like NK cells; TCR, T-cell receptor; CAR, Chimeric antigen receptor; CTLs, Cytotoxic T-lymphocytes.

Numerous scientific advances have contributed to the resurgence of haplo-HCT as a viable transplant option for patients requiring HCT and have achieved similar outcomes to those from other donor sources. The ability to perform haplo-HCT without costly *ex-vivo* T-cell depletion approaches, which require extensive cell manufacturing expertise frequently limited to large transplant centers, has been a major advance in transplant accessibility for patients in resource-limited countries that frequently do not perform unrelated donor transplantation (203). However, further efforts are required to improve immune reconstitution, control infectious complications and decrease relapse rates in patients after haplo-HCT. Fortunately, haplo-HCT provides an ideal platform characterized by unique immunologic properties and ready accessibility of the donor for additional cell products. This offers tremendous opportunities for the development and implementation of innovative adoptive immune cell therapies to augment infectious and antitumor immunity and further improve outcomes (Figure 9).

### Suicide Mechanisms for Defined T-Cell Content in the Graft and Post-transplant

*Ex-vivo* TCD haplo-HCT affords opportunities not only for the dose-titration but also the manipulation of the T cell product prior to infusion into the patient. Rather than *in-* or *ex-vivo* approaches to selectively deplete or attenuate T cells, a different approach is the infusion of polyclonal T cells that have been genetically engineered to include an inducible suicide gene. With this strategy, a defined dose of T cells can be administered to aid in engraftment and immune reconstitution, mediate a GVL effect, and provide infectious immunity while being selectively susceptible to an externally inducible suicide mechanism in the event of significant GVHD (204).

The first such approach was pioneered by Bonini and colleagues with the introduction of a herpes simplex virus thymidine kinase (HSV-TK) suicide gene into T cells using γ-retroviral transduction in which the transgene also contained the truncated selection marker ΔLNGFR. This allowed for the isolation and infusion of transduced cells bearing the suicide gene (205). With this strategy, administration of the drug ganciclovir activated the suicide mechanism and successfully controlled GVHD in several patients after infusion (204). Interestingly, the first wave of circulating TK^+^ cells after infusion facilitated thymic renewal and was followed by a second wave of long-term immune reconstitution with naïve lymphocytes. This was supported by an increase in TCR excision circles, CD31^+^ recent thymic emigrants and expansion of thymic tissue on imaging and was further associated with an increase in serum IL-7 levels following each infusion (206).

Since then, other approaches have been developed, such as transduction of T-cells with the iCasp-9 suicide gene. This gene can be activated by an otherwise inert drug (207, 208). Novel approaches have also included the use of a different transduction marker such as truncated CD19 that allows for the confirmation of transduction and if desired positive isolation of transduced T cells prior to infusion. Brenner and colleagues first utilized this approach in children undergoing haplo-HCT and demonstrated impressively how iCasp-9 transduced T cells expressing the truncated CD19 aid in immune reconstitution and contribute to infectious immunity (207, 208). Activation of the suicide gene led to resolution of GVHD symptoms within hours (209, 210). Interestingly, while alloreactivity was rapidly abrogated, suicide-gene transduced T cells were not permanently eliminated and able to reconstitute again without causing GVHD. Pediatric studies are underway to investigate suicide-gene equipped T-cell infusions after αβ-TCR/CD19 depleted haplo-HCT.

### Haploidentical Donor Lymphocyte Infusions

A common approach to address relapse early after HCT is the infusion of donor lymphocyte infusions (DLI) to exert a GVL effect, but this is frequently accompanied by significant rates of GVHD. Zeidan and colleagues demonstrated the feasibility of this approach after haplo-HCT with PTCy in a retrospective analysis of a dose escalation approach at their center (211). Forty patients received 52 haplo-DLI doses initially at 1 × 10^5^ CD3^+^/kg and most commonly starting at 1 × 10^6^ CD3^+^/kg. Ten patients (25%) developed GVHD with Grade III-IV acute GVHD in 6 and chronic GVHD in 3 patients. Twelve patients (30%) achieved a CR with a median duration of 11.8 months (211).

Sun et al. reported on haplo-DLI following a number of different chemotherapy regimens (FLAG, Methotrexate and others) for relapse after haplo-HCT with the GIAC protocol. Of 86 patients, 20 developed Grade III-IV aGVHD and 41 developed cGHVD. NRM was 10.3%, and 62% of patients achieved a CR after chemo-DLI of which 50% experienced re-relapse at a median duration of 92 days (212). A modified GIAC backbone was also utilized to assess preemptive DLI at a median of 77 days post haplo-HCT in high risk patients to prevent relapse. With a sizeable median CD3^+^ dose of 1.8 × 10^7^/kg, the 100-day incidences of acute GVHD were 55.3% for Grade II–IV and 10.2% for Grade III–IV, respectively. Two-year incidence of chronic GVHD was 52%, among which 18.2% were severe. With this regimen, 2-year NRM was high at 33.1% with a 2-year relapse incidence of 32% (213). Approaches to reduce GVHD while optimizing the GVL effect of preemptive or therapeutic DLI are likely to evolve over time and include the infusion of IL-10 anergized DLI (214), CD45-RA depleted DLI (215) and adoptive transfer of gene modified cells as described in this section. Although experience in the haplo-HCT setting is limited to date, azacitidine or decitabine in conjunction with DLI have shown promising overall response rates on the order of 25–33% for patients with AML or MDS relapsing after allogeneic HCT (216, 217).

### CAR- T or CAR-NK-Cell Infusion

Chimeric antigen receptor (CAR) T cells targeting CD19 have revolutionized the treatment of relapsed/refractory B-cell acute lymphoblastic leukemias and aggressive B-cell lymphomas, with complete remission rates ranging from 70–90% in ALL (218, 219) and ~60% for refractory large B-cell lymphoma (220, 221). CAR-T cells have been successfully manufactured from donor T cells in patients with relapse after allogeneic HCT and infused without mediating GVHD. Autoimmune complications have not been observed after infusion of CAR-T cells derived from autologous T cells suggesting that the CAR-signal overrides TCR-based recognition. The use of third-party CAR-T cells has been explored with concurrent transcription activator-like effector nuclease (TALEN)-based gene editing of the endogenous TCR. These CAR-T cells did mediate GVHD in a limited study of three patients (222). The use of CAR-NK cells is also being explored in the relapse setting, and although long-term persistence may be more limited than that of CAR-T cells, this approach may be beneficial when there is a higher degree of HLA-mismatch such as after haplo-HCT (223). While the therapeutic success of CD19-targeting CAR-T cell therapy to date is limited to B-cell malignancies and multiple myeloma (224), studies are underway to investigate the safety, feasibility, and preliminary efficacy of CAR-T cells directed against AML and MDS (225–227). Given this rapidly evolving field, the established efficacy potential of CAR-T cells and ability to utilize donor cells for CAR-T cell manufacture post-HCT, haploidentical HCT donors represent a readily available post-transplant cell source for donor-derived CAR-T cell or CAR-NK cell therapies for relapsed leukemia.

### Antiviral Cytotoxic T Lymphocyte (CTL) Infusion

Infectious complications and particularly end-organ viral disease after HCT remain a challenge, particularly in haplo-HCT where *ex-* or *in*- vivo T-cell depletion is necessary. For example, the incidence of BK-virus hemorrhagic cystitis is higher in haplo-HCT (228). As demonstrated by Leen and Bollard the infusion of virus-specific CTL lines, generated by stimulating PBMC from adenovirus and EBV-seropositive donors, can safely be performed without inducing GVHD and can result in clearance of adenoviral disease and prevention of EBV-associated PTLD (229). The successful use of off-the-shelf multi-virus-specific T cells to treat viral infections after allogeneic HCT with minimal risk of GVHD has since been confirmed in a larger study and has the potential to mitigate serious viral disease after haplo-HCT either with third-party or haploidentical antiviral CTLs (230).

### TCR-Edited T Cell Infusions

Whereas, CAR-transduced T cells recognize extracellular peptides on the surface of target cells in an MHC-independent manner, TCR-mediated T cell recognition mediates T cell immunity against MHC-restricted, intracellular targets and minor histocompatibility antigens. With the advent of sophisticated strategies to optimize T cell transduction and prevent mis-coupling of transduced and endogenous TCR chains, TCR-edited T cells have successfully entered clinical trials for patients with an HLA-type required for the HLA-restricted expression of the antigen. Greenberg and colleagues cloned a high affinity TCR targeting the HLA-A2 restricted tumor antigen WT-1 from healthy donors and inserted this TCR into EBV-specific donor CD8^+^ T cells (to minimize the GVHD risk and enhance persistence). The WT1-TCR modified donor T cells were then infused prophylactically into the HLA-A^*^0201+ recipients after they had received an allogeneic HCT from the same donor. This approach resulted in 100% relapse free survival in the WT-1 TCR-T cell group at 44 months as compared to a comparative group of similar risk AML patients with a 54% relapse-free survival after HCT (231). A separate approach is currently under investigation to target the HLA-A^*^0201-restricted minor histocompatibility antigen HA-1, which is exclusively expressed on hematopoietic cells (232). When the immunogenic single-nucleotide polymorphic variant of HA-1 is expressed on hematopoietic cells of the HLA-A2+HCT-recipient, donor T cells that have been transduced to encode a high-avidity TCR recognizing HA-1 can effectively eliminate leukemia and lymphoma cells *in vitro* (233). Given the facile availability of donor T cells, haplo-HCT can and should serve as a beneficial platform to explore new approaches to reduce relapse after HCT.

### NK Cell Product Infusion to Augment Graft vs. Tumor Effect

As previously described, NK-cells can mediate GVL effects due to KIR-mediated alloreactivity in the haplo-HCT setting. In addition to selecting the donor based on predicted NK-cell alloreactivity, the availability of haploidentical donors for additional cell product collection affords the unique opportunity to utilize NK-cell infusions to provide for additional GVL or GVT effects after HCT prophylactically or in the face of relapse (234). Generation of adequate numbers of NK cells for post-transplant therapies can be challenging given the relatively low NK cell frequency in the blood but can be overcome by *in vitro* expansion such as with membrane-bound IL-21 expressing feeder cells (mbIL21). A Phase 1 study evaluated prophylactic NK cell infusions after haplo-HCT with PTCy on days −2, +7, and +28. Of 11 enrolled patients who received all 3 planned NK cell doses, 54% developed Grade I-II aGVHD, and none developed Grade III-IV aGVHD, chronic GVHD or dose-limiting toxicities. Only 1/11 patient relapsed. All others were alive and in remission at a median follow-up of 14.7 months (235). Administration of cytokines can facilitate NK cell expansion, but certain cytokines such as IL-2 also preferentially expand Tregs based on their constitutive expression of high-affinity IL-2R (CD25). These Tregs in turn inhibit NK cell proliferation (236). A study treating AML patients with haploidentical NK cell infusions after lymphodepletion with cyclophosphamide and fludarabine demonstrated that NK cell expansion was most pronounced and effective when IL-2-diphteria toxin fusion protein was administered to achieve host Treg depletion (237).

A recent trial administering haploidentical NK cells with rhIL15 for relapsed AML after lymphodepleting chemotherapy showed that rhIL-15 achieved better rates of *in vivo* NK-cell expansion and remission compared to previous trials utilizing IL-2, but also observed steroid- and tocilizumab-responsive cytokine release syndrome and neurologic toxicity which was associated with high levels of IL-6 (238). Cytokine-induced memory-like (CIML) NK cells from haploidentical donors were able to induce complete remissions in relapsed/refractory AML patients outside of the transplant setting without any toxicities (239). This GVL effect may be even more durable when NK cells from the same haploidentical donor are infused after haplo-HCT because no immunologic rejection of the CIML NK cells from the same donor is expected. Studies to date have suggested that KIR-reactivity is less important when NK cells are cytokine-induced (240). Studies are now underway to evaluate the safety and efficacy of CIML NK cells for relapse after haplo-HCT.

### Cytokine Support to Enhance NK-Cell Alloreactivity After Hct

An alternative strategy to address relapse after HCT is the administration of cytokines aimed at enhancing the anti-leukemic function of the existing post-transplant immune environment. One such approach employed ALT-803, an IL-15 superagonist complex designed to extend the *in vivo* half-life of IL-15 and mimic the physiologic *trans*-presentation of IL-15 (241). In contrast to IL-2 that can promote the survival, proliferation, and activation of lymphocytes, but that also stimulates Tregs, IL-15 preferentially expands CD8^+^ T cells and NK cells via trans-presentation to the IL-2/15Rβγ_c_-receptor while avoiding the stimulation of Tregs. In a recent Phase 1 trial ALT-803 was well-tolerated, particularly when administered subcutaneously, and induced responses of 19% in patients relapsed after HCT (241), suggesting that such agents may also be explored in the haplo-HCT setting. Efforts are underway to test use of IL-15 or IL-15 superagonist complex alone or in combination with NK cell- based therapy to target relapse after haplo-HCT.

## Conclusion

The initial immunologic barriers to haplo-HCT, namely GVHD and graft failure, have been overcome with different platforms that can be utilized to control T cell alloreactivity post-transplant. Comparable clinical outcomes have now been achieved relative to alternative donor sources and depending on the specific scenario, haplo-HCT can offer a lower risk of GVHD and/or improved control against relapse. The GVL effect in haplo-HCT is particularly intriguing given the concept of NK-cell alloreactivity based on the KIR/KIR-ligand system and ability to select donors accordingly. An emerging body of literature is elucidating immunologic mechanisms of GVHD and relapse that are potentially targetable and highlight the immune pressure exerted by donor immune cells after HCT. Given ready accessibility of the donor, haplo-HCT offers a unique platform for post-transplant cell-based immune therapies aimed at expediting immune reconstitution, improving thymic function, providing infectious immunity, and treating or protecting against relapse, while maintaining therapeutic control of those cell immunotherapies with methods such as suicide mechanisms. The rapid advancements in our understanding of the immunobiology of haplo-HCT are therefore poised to lead to increasingly sophisticated strategies to fine-tune the transplant process and to further improve outcomes after haplo-HCT.

## Author Contributions

SB, BR, RS, and RR helped review the literature and wrote this manuscript.

### Conflict of Interest

The authors declare that the research was conducted in the absence of any commercial or financial relationships that could be construed as a potential conflict of interest.
